# The DNA integrity number and concentration are useful parameters for successful comprehensive genomic profiling test for cancer using formalin‐fixed paraffin embedded tissue

**DOI:** 10.1111/pin.13318

**Published:** 2023-03-27

**Authors:** Emmy Yanagita, Hiroshi Yamada, Tetsuro Kobayashi, Eriko Aimono, Kohei Nakamura, Akira Hirasawa, Hiroshi Nishihara

**Affiliations:** ^1^ The Department of Clinical Laboratory Keio University Hospital Tokyo Japan; ^2^ Clinical Genomic Medicine Okayama University Graduate School of Medicine, Dentistry and Pharmaceutical Science Okayama Japan; ^3^ Genomics Unit, Keio Cancer Center Keio University School of Medicine Tokyo Japan; ^4^ School of Public Health Kyoto University Kyoto Japan

**Keywords:** clinical sequence, DNA concentration, DNA integrity number, DNA quality, formalin‐fixed paraffin embedded tissue, next generation sequencer

## Abstract

The acquisition of high‐quality biospecimens and the appropriate handling of these materials are indispensable for successful clinical sequencing. We developed a cancer clinical sequencing system targeting 160 cancer genes: PleSSision‐Rapid. Through the PleSSision‐Rapid system, we have analyzed DNA quality evaluated by DIN (DNA integrity number) with 1329 formalin‐fixed paraffin embedded (FFPE) samples including 477 prospectively collected tissues for genomic test (P) and 852 archival samples after routine pathological diagnosis (A1/A2). As a result, the samples with more than DIN 2.1 was 92.0% (439/477) in prospectively collected sample (P), while it was 85.6% (332/388) and 76.7% (356/464) in two types of archival samples (A1/A2). We performed the PleSSision‐Rapid sequence using the samples with over DIN 2.1 and DNA concentration >10 ng/μL with which we were able to construct a DNA library, and the probability of sequence success was almost equivalent during all types of specimen processing, at 90.7% (398/439) in (P), 92.5% (307/332) in (A1) and 90.2% (321/356) in (A2), respectively. Our result indicated the clinical benefit to prepare the prospective collection of FFPE materials for indisputable clinical sequence, and that DIN ≥ 2.1 would be a solid parameter for sample preparation of comprehensive genomic profiling tests.

**Abbreviations**
CGP: comprehensive genomic profiling
DIN: DNA integrity number
FFPE: formalin‐fixed paraffin embedded
PleSSision: Pathologists edited, Mitsubishi Space Software supervised clinical sequence system for personalized medicine

## INTRODUCTION

Development of cancer genomics driven by next generation sequencing technologies enables us to perform contemporary clinical sequencing using formalin‐fixed paraffin embedded (FFPE) specimens which are prepared for routine pathological diagnosis. FoundationOne^1^ and NCC oncopanel^2^ are the leading clinical sequencing services in Japan for all types of cancer patients, and both genomic tests officially employed FFPE section. The quality control for the sequence is essential for the successful sequencing; however, the definite criteria for sample quality have not been demonstrated. Previously, the Japanese Society of Pathology published guidelines on the handling of pathological tissue samples for genomic research^3^ and mentioned the significance of DIN (DNA integrity number) based on the experimental data. DIN has been discussed already in correlation with the DNA amount and DNA concentration for the successful library prep,^4^, ^5^ but the real‐world clinical data was not revealed.

In Keio University Hospital, we have performed an in‐house clinical sequence study for variable types of cancer and collected more than 2000 samples as prospective and archival sample collection under the routine surgery‐pathological examination pathway. Through this clinical sequence study, we evaluated the sample quality correlated with successful sequencing, and propose the clinical value of DIN and DNA concentration as a criterion for the clinical sequence.

## MATERIALS AND METHODS

### Ethical issue

This study was approved by the ethics committee of Keio University (approval number: 20180015) and was conducted in accordance with the Declaration of Helsinki and Title 45, U.S. Code of Federal Regulations, Part 46, Protection of Human Subjects, effective December 13, 2001. All patients provided written informed consent.

### Patients’ surgical material and tissue fixation

The surgical material came from all patients who underwent surgical resection including excisional biopsy for solid tumor in Keio University Hospital from April 2017 to March 2020. The samples were obtained during the surgery (prospective collection) or after the pathological examination (archival collection), and the types of formalin for fixation of the surgical material differed depending on the surgery department. The departments of breast surgery, thoracic surgery and hepatobiliary/pancreatic surgery used 10% buffered formalin, and the other departments used 20% buffered formalin.

The prospective collection samples (P) resected during the surgery were immediately fixed by 10% buffered formalin and embedded to paraffin within 24 h. The archival samples were obtained after the pathological diagnosis and categorized into the following two groups: A1 was fixed by 10% formalin and A2 was fixed by 20% formalin. The fixation time of the archival samples varied depending on the business procedure of each department, and most of the departments processed the sample within 72 h but, as an exception, the department of hepatobiliary/pancreatic surgery finalized the formalin fixation at around 7 days. The number and attributes of the samples are listed in Tables 1 and 2.

**Table 1:** Summary of the sample; DNA concentration and DNA integrity number (DIN).

					The breakdown of DIN ≧ 2.1		
Organ	Sample number	Low DNA conc.	DIN < 2.1	DIN ≥ 2.1	2.1 ≤ DIN < 3.0	3.0 ≤ DIN < 4.0	4.0 ≤ DIN
*P; prospective collected sample*							
Breast	5	0	0	5	0	0	5
Lung	30	0	0	30	4	6	20
Hepatobiliary and pancreas	14	0	3	11	3	2	6
Head and neck	4	0	0	4	0	1	3
Colorectal	50	0	0	50	9	3	38
Brain	37	0	2	35	3	5	27
Gynecology	109	0	9	100	15	28	57
Oral	22	1	2	19	3	6	10
Skin	9	0	0	9	2	2	5
Urology	129	5	9	115	29	11	75
Soft tissue and bone	29	1	0	28	2	4	22
Esophagus and stomach	39	2	4	33	8	5	20
Total	477	9/477(1.9%)	29/477 (6.1%)	439/477 (92.0%)	78/477 (16.4%)	73/477(15.3%)	288/477 (60.4%)
*A; archival sample*							
A1							
Breast	302	18	19	265	55	99	111
Lung	49	0	5	44	12	12	20
Hepatobiliary and pancreas	37	0	14	23	10	10	3
Total	388	18/388(4.6%)	38/388 (9.8%)	332/388 (85.6%)	77/388 (19.8%)	121/388 (31.2%)	134/388 (34.5%)
A2							
Head and neck	6	0	1	5	1	3	1
Colorectal	86	0	20	66	41	22	3
Brain	21	1	1	20	4	8	8
Gynecology	147	4	35	108	50	33	25
Oral	3	0	0	3	1	2	0
Skin	14	0	2	12	3	3	6
Urology	62	1	11	50	13	21	16
Soft tissue and bone	20	6	8	6	4	1	1
Esophagus and stomach	105	2	17	86	31	38	17
Total	464	14/464(3.0%)	94/464 (20.3%)	356/464 (76.7%)	148/464 (31.9%)	131/464 (28.2%)	77/464 (16.6%)
Total (A1＋A2)	852	32/852(3.6%)	132/852 (15.5%)	688/852 (80.8%)	225/852 (26.4%)	252/852 (29.6%)	211/852 (24.8%)

**Table 2:** Summary of sequence result according to DNA concentration and DNA integrity number (DIN).

				DIN ≥ 2.1		
Organ	Sample number	Low DNA conc. (<10 ng/μL)	DIN < 2.1	Number of Sequencing	Seq. success case	Sequence error
*P; prospective collected sample*						
Breast	5	0	0	5	5	0
Lung	30	0	0	30	30	0
Hepatobiliary and pancreas	14	0	3	11	9	2
Head and neck	4	0	0	4	4	0
Colorectal	50	0	0	50	46	4
Brain	37	0	2	35	33	2
Gynecology	109	0	9	100	95	5
Oral	22	1	2	19	19	0
Skin	9	0	0	9	8	1
Urology	129	5	9	115	96	19
Soft tissue and bone	29	1	0	28	25	3
Esophagus and stomach	39	2	4	33	28	5
Total	477	9/477(1.9%)	29/477 (6.1%)	439/477 (92.0%)	398/477 (83.4%; in total) 398/439 (90.7%; in DIN ≧ 2.1)	41/477 (8.6%) 41/439 (9.4%)

				DIN ≧ 2.1		
Organ	Sample number	Low DNA conc.	DIN < 2.1	Number of Sequencing	Seq. success case	Sequence error
*A; archival sample*						
A1						
Breast	302	18	19	265	241	24
Lung	49	0	5	44	43	1
Hepatobiliary and pancreas	37	0	14	23	23	0
Total	388	18/388(4.6%)	38/388 (9.8%)	332/388 (85.6%)	307/388 (79.1%; in total) 307/332 (92.5%; in DIN ≧ 2.1)	25/388 (6.4%) 25/332 (7.5%)
A2						
Head and neck	6	0	1	5	5	0
Colorectal	86	0	20	66	58	8
Brain	21	1	1	20	19	1
Gynecology	147	4	35	108	95	13
Oral	3	0	0	3	3	0
Skin	14	0	2	12	12	0
Urology	62	1	11	50	47	3
Soft tissue and bone	20	6	8	6	6	0
Esophagus and stomach	105	2	17	86	76	10
Total	464	14/464 (3.0%)	94/464 (20.3%)	356/464 (76.7%)	321/464 (69.2%; in total) 321/356 (90.2%; in DIN ≧ 2.1)	35/464 (7.5%) 35/356 (9.8%)
Total (A1＋A2)	852	32/852 (3.6%)	132/852 (15.5%)	688/852 (80.8%)	628/852 (73.7%; in total) 628/688 (91.3%; in DIN ≧ 2.1)	60/852 (7.0%) 60/688 (8.7%)

### DNA extraction and quality examination

In our system, genomic DNA was extracted from FFPE tissue derived from biopsy specimen (15 slides of 10 μm‐thick slices) and surgically resected cancer tissue (five slides of 10 μm‐thick slices). We evaluated the DNA quality of extracted DNA using DIN (DNA integrity number) measured by TapeStation 4200 (Agilent Technologies) which indicated the degree of DNA fragmentation.^3^

### Target amplicon sequence

Genomic DNA, which was extracted from thin sliced FFPE tissue using the Maxwell RSC Instrument（Promega), was applied for library construction with the GeneRead DNAseq Targeted Panels V2 (Human Comprehensive Cancer Panel), which covers more than 95% area of exon region in 160 cancer‐related genes (Supporting Information: Supplementary Table 1). Multiplex PCR and purification were performed with the GeneRead DNAseq Panel PCR Kit V2 (QIAGEN) and AgencourtAMPure XP Beads (BECKMAN COULTER), followed by measurement of total DNA amount by Qubit 4.0 Fluorometer dsDNA HS assay kit (ThermoFisher SCIENTIFIC). We performed initial screening for DNA quality using TapeStation 4200, and we obtained the DIN (DNA integrity number). End repair and adaptor‐ligation were performed by GeneRead DNA Library I core kit (QIAGEN). The library was amplified using GeneRead DNA I Amp kit (QIAGEN). We tried to construct the DNA library with all samples but could not obtain the sufficient amount for the DNA library with DIN < 2.1 and/or low DNA concentration (<10 ng/μL) samples, meaning disability of sequence. The pooled library was sequenced by NextSeq 550 (Illumina).

### Data analysis

The FastQ files obtained from NextSeq were analyzed using an original bioinformatics pipeline called GenomeJack (Mitsubishi Space Software) (http://genomejack.net/). In brief, sequenced reads were mapped with BWA 0.7.12,^6^ and realigned with abra 0.97.^7^ The sequence reads of <75 bps or QV < 30 were filtered out, and if the residual reads were <70%, we called them “sequence error.” We categorized DIN < 2.1 and/or low DNA concentration (<10 ng/μL) cases (not applied for sequence), and “sequence error” into the “Failure” group, and the others into the “Success” group. We identified somatic gene alterations such as single nucleotide variations (SNVs), insertions (Ins)/deletions (Del), and copy number variations. For identification of SNVs, samtools was employed to count the sequence reads,^8^ and the defective reads with <10 counts and also the SNVs with confliction between multiple amplicons were abandoned. In addition, SNVs with <5% of variant allele frequency (VAF) were ignored as possible sequence error. Ins/Del was detected by a modified version of VarScan 2.3.9,^9^ and the cut‐off value was set as *p* < 0.05. Possible germ line single nucleotide polymorphisms (SNPs) were excluded using dbSNP, Human Genetic Variation Database (HGVD) and the database of ToMMo 2KJPN (Tohoku Medical Megabank Organization). The copy number of each gene was calculated as a mean value of all reads covering the target gene and compared with the average of the result of control samples.

### Receiver operating characteristics (ROC) curves

We used 1329 (477 + 852) samples, with known values for DIN and DNA concentrations used at the time of sequencing, to determine the cut‐off values as a predictor of sequence success. Of the samples, sequence was performed in 1127 (439 + 688) samples with DIN ≥ 2.1 and was successful in 1026 (398 + 628) samples and ended up in failure in 101 (41 + 60) samples. When we analyzed the detailed DIN and sequence success rate, DIN > 3.0 samples revealed the apparent high success rate (Supporting Information: Supplemental Figure 1). We then calculated the sensitivity and specificity at differing cut‐off values of DIN, DNA concentrations, and combination of both. We set the cut‐off values of 2.1, 2.5, 3.0, 3.5, 4.0, and 5.0 for DIN and 10, 20, 30, 40, 50, 150, and 200 (ng/μL) for DNA concentrations. Of note, samples with DIN values of <2.1 were discarded because of failure for DNA library construction and thus not included in the analysis. We drew ROC curves using DIN alone, using DNA concentrations alone, and in combinations of both. For the ROC curves using both DIN and DNA concentrations in combinations, we set a fixed a value for either DIN or DNA concentrations at a certain value and used differing values of the other to calculate the sensitivity and specificity of such parameter settings. We determined the cut‐off values of DIN and DNA concentrations that gave the maximum area under the curve (AUC) of the ROC.

## RESULTS

**Figure 1:**
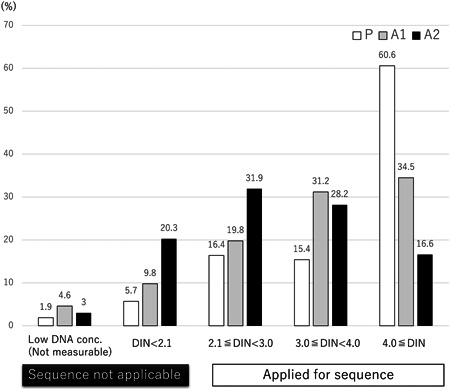
Specimen attributes and the incidence of sequence error. The range of DNA integrity number (DIN) was indicated according to the method of specimen processing. In the prospectively collected samples (P), the proportion of high quality sample (4.0 ≤ DIN) was over 50%, overwhelmingly higher than that of archival samples (A1, A2), meaning that suitable sample processing using 10% formalin was a feasible factor for high quality sample.

**Figure 2:**
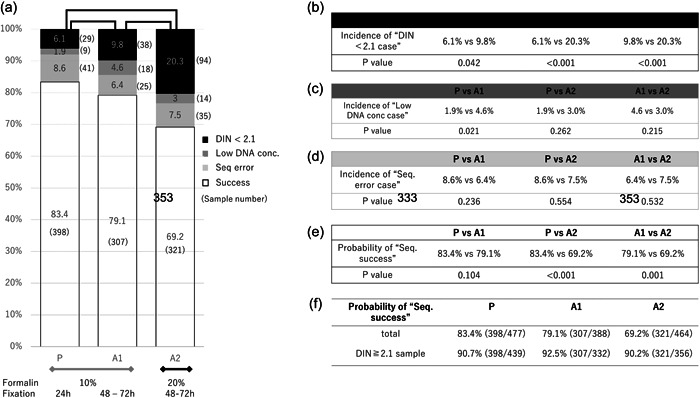
Probability of “Seq. success” of FFPE samples according to the primary organs. The samples were obtained during the surgery (prospective collection: P) or after the pathological examination (archival collection: A1 and A2). The sample group (P) was fixed by 10% buffered formalin and embedded to paraffin within 24 h, while A1 was fixed by 10% formalin and A2 was fixed by 20% formalin for 48–72 h. The incidence of “DIN < 2.1,” “Low DNA conc. <10 ng/μL” and “Seq. error” was comparatively analyzed in each group as we as probability of “Seq. success” (b–e). The probability of “Seq. success” excluded “DIN < 2.1” and “Low DNA conc. <10 ng/μL” was also comparatively analyzed (f). By Bonferroni's correction, we set the significance level at 0.017 instead of the conventionally used value of 0.05. DIN, DNA integrity number; FFPE, formalin‐fixed paraffin embedded.

We first compared the quality of DNA belonging to three groups (Table 1 and Figure 1). The ratio of high DNA quality sample (DIN ≥ 4.0) was over 50% in (P) group and obviously higher than those in the archival sample groups (A1 and A2). The incidence of DIN < 2.1 sample was significantly lower in (P) compared with (A1) and (A2) (Figure 2a, b). In addition, the (A2) samples revealed the significantly higher incidence of low DIN samples compared with (A1) (Figure 2a, b). The fixation time in (A1) and (A2) was more than 48 h and longer than in (P: 24 h), and the 20% formalin was employed in (A2). These results indicated that the longer and stronger fixation by formalin would result in worse DIN as shown in previous reports.^10^, ^11^ Meanwhile, the proportion of the samples with low DNA concentration, which were unmeasurable for DIN, was similar among the three groups because of insufficient tissue size (Figure 2a, c).

We excluded the samples of DIN < 2.1 for sequence because we failed to construct the DNA library, and this result was preconceived by the previous study which revealed DIN ≥ 2.1 as the critical cut‐off for DNA quality.^12^ The samples with low DNA concentration (<10 ng/μL) were also excluded because of the technical difficulty to input the adequate amount of DNA for library construction. The probability of successful sequence among total samples was significantly higher in (P) and (A1) groups compared with (A2) (Figure 2e), whereas, if limited to the samples with over DIN 2.1, it was almost equivalent, at 90.7% (398/439) in (P), 92.5% (307/332) in (A1) and 90.2% (321/356) in (A2), respectively (Figure 2f). This result revealed that the successful clinical sequence is simply dependent on DNA quality, thus the preparation of high DNA quality FFPE specimen is crucial regardless of fixation methods. In a similar matter, the incidence of sequence error was similar among three groups (Figure 2d).

**Figure 3:**
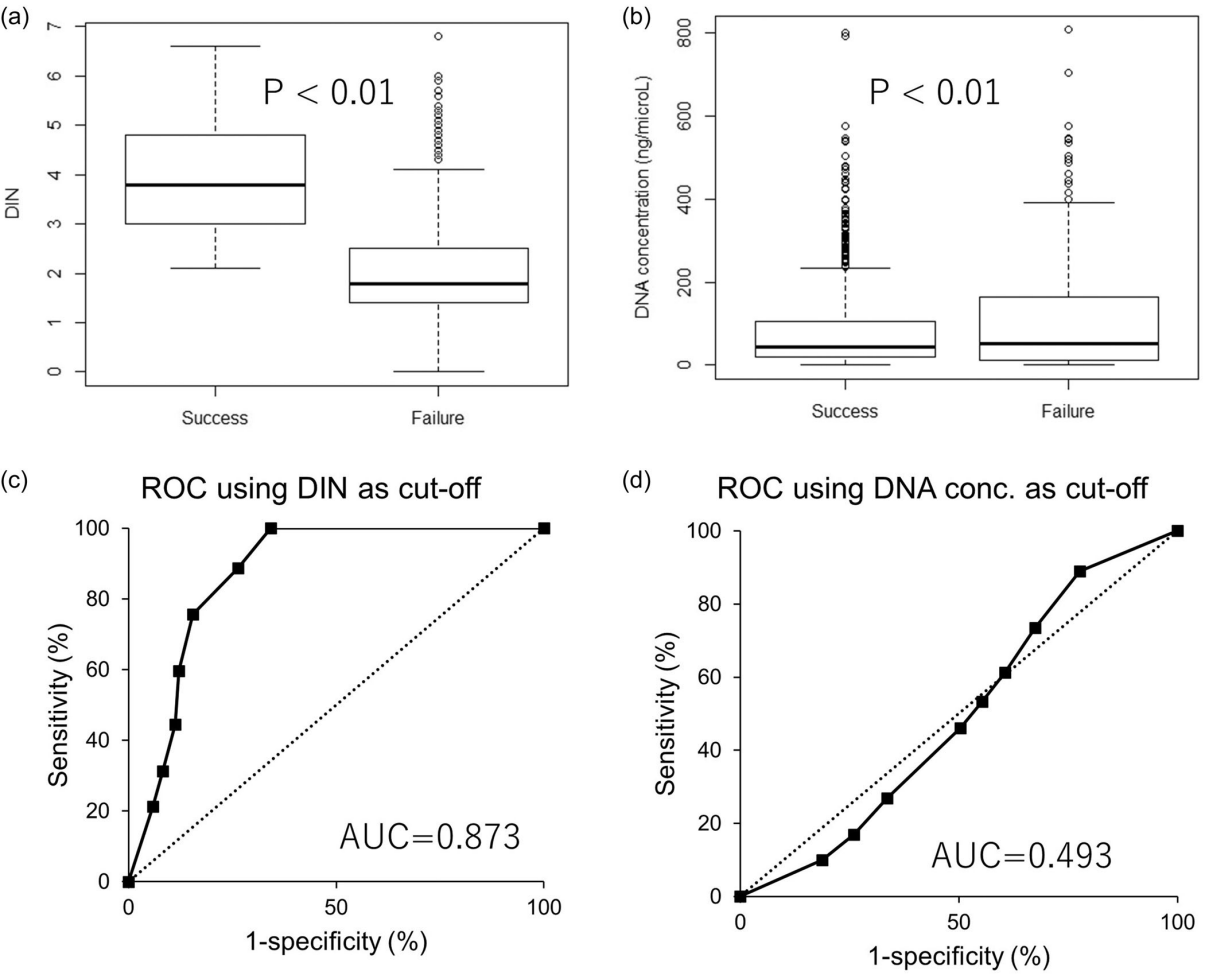
The significance of DIN and DNA concentration as sequence parameters. Comparison of DIN (a) and DNA concentration (b) with respect to sequence result. We categorized DIN < 2.1 and/or low DNA concentration <10 ng/μl cases (not applied for sequence), and “sequence error” into the “Failure” group, and the others into the “Success” group. *P*‐values: Welch's *t*‐test was performed with respect to the sequencing success. ROC curves: DIN (c) or DNA concentration (d) is used as a single predictor for successful sequencing. DIN, DNA integrity number; ROC, receiver operating characteristics.

Next, we evaluated the DIN and DNA concentration in correlation with sequence success rate. As shown in Figure 3a, when Welch's *t*‐test was performed, DIN was significantly higher in sequence success samples compared with failure samples, while DNA concentration was paradoxically higher in failure samples (Figure 3b). Looking at the details of the sample distribution, “Success” samples are obviously distributed in high‐DIN area compared with the “Failure” sample (Supporting Information: Supplemental Figure 2, left panel). Meanwhile, the sample distribution according to DNA concentration in “Success” and “Failure” cases was almost identical, although the failure group is slightly skewed to the right, leading to the elevation of the DNA concentration in the failure group (Supporting Information: 2, right panel). When DIN was used alone as a single predictor for successful sequencing, the ROC curve gave the AUC of 87.3%; the cut‐off level of 3.0 gives sensitivity of 75.7% and specificity of 84.5% (Figure 3c and Supporting Information: supplemental Table 2). On the other hand, when DNA concentration was used as a predictor, the ROC curve gave the AUC of 49.3%; the cut‐off level of 40 ng/μL gives sensitivity of 53.3% and specificity of 44.7% (Figure 3d and Supporting Information: Supplemental Table 2), indication that DNA concentration could not be a useful predictor for successful sequence. When both DIN and DNA concentrations were used in combination, the cut‐off criteria with DNA concentration fixed at 10 ng/μL gave the AUC of 86.7% (Supporting Information: Supplemental Figure 3), in which the DIN cut‐off level of 2.1 gave a sensitivity of 89.0% and specificity of 74.7%. When DNA concentration cut‐off level was fixed at 20 ng/μL, the AUC was 80.0%, but the DIN cut‐off level of 2.1 gave the sensitivity of 73.5% and specificity of 78.9%. The dot plot of the individual sequence result revealed that the area of DIN > 3.0 and DNA conc. >150 ng/μL means the guarantee of indisputable sequencing (Supporting Information: Supplemental Figure 4); however, the sensitivity was extremely low (9.3%, Supporting Information: Supplemental Table 3). In the clinical setting, the clinical sequence would be performed with the archival FFPE specimen such as in (A1/A2), and >40% of them revealed the low DIN of <3.0 (Table 1 and Figure 1), and sometimes it would be hard to obtain the fresh tissue from such patients. Therefore, the strict cut‐off value of such as DIN > 3.0 might make >20% false‐negative cases (Supporting Information: Supplemental Table 2).

## DISCUSSION

Throughout our study, we confirmed the apparent clinical benefit to prepare FFPE specimen with minimum DIN ≥ 2.1 for successful clinical sequence based on real‐world data using more than 1300 FFPE samples. Meanwhile, DNA concentration >10 ng/μL would be the minimum requirement for DNA library construction but not a significant predictor for successful sequence. We calculated the positive prediction value for successful sequencing based on DIN and DNA concentration (Table 3) and it would be useful to decide whether to implement sequence with the sample by predicting the failure risk and possibility of getting the valuable sequence report. However, in case of the sample with DIN < 3.0, we recognized that higher DNA conc. conversely led to lower success rate. It might depend on the inhibitory effect of fragmented DNA to waste the sequence probe during the library construction. Our research would be the first report to present the correlation between DIN and DNA concentration and the positive prediction value of DIN and DNA concentration for the successful sequence with the statistical evidence.

**Table 3:** Positive predictive value for successful sequencing.

	DNA conc.							
DIN	10	20	30	40	50	100	150	200
2.1	92.1	92.1	92	92.6	92.1	90.7	88.1	85.6
2.5	93.5	93.7	93	93.5	92.9	91.6	89.1	85.9
3	96.5	97.6	97.7	98.1	97.7	98	98.9	100
3.5	97.1	98	98.4	98.5	98.2	98.2	98.5	100
4	96.4	97.2	97.7	97.8	97.2	97.3	97.6	100
4.5	96.5	96.9	97.3	97.5	96.8	98	100	100
5	97.2	96.9	97	97.5	97	97.4	100	100

Type of the formalin and the adequate fixation time, with the adjustment depending on the organ characteristics in each case, are the credible parameters for high quality FFPE specimens. Considering the quality control for the successful sequence in clinical setting, the definite parameters for quality management will be required. We reviewed the incidence of DIN < 2.1 sample according to the primary organs. Interestingly, the (P) and (A1) samples showed the favorable results in most cases except for the sample derived from the department of hepatobiliary/pancreatic surgery in which 3 out of 14 cases in (P) (21.4%) and 14 out of 37 cases in (A1) (37.8%) were less DIN ≤ 2.1 (Table 1). The (P) sample was supposed to be trimmed from the surgically resected tissue and fixed in formalin as soon as possible, thus we expected the highest DNA quality, even in the case of the department of hepatobiliary/pancreatic surgery. We hypothesized two reasons for this result: one is simply protocol deviation such as the longer abandoning of the tissue until the trimming, and the other is the organ characteristic. The samples from the department of hepatobiliary/pancreatic surgery were liver, gallbladder, and pancreas; these contain plentiful digestive enzymes such as lipase and protease, thus the longer abandon time will permit autolysis before formalin fixation.

The sequence platform was varied in each comprehensive genomic profiling (CGP) test; however, the minimum requirement DNA quality for a successful sequence would not be much different. Among our research participants, nine patients subsequently underwent FoundationOne CDx as a reimbursed CGP test using the same FFPE tissue. The test was successfully performed in all of the nine cases including six cases of DIN ≥ 2.1 and three cases of DIN < 2.1. This result indicated that the reimbursed CGP test could be performed even with the sample of DIN < 2.1 but DIN ≥ 2.1 would be one of sufficient conditions for successful sequence. Based on these results, the best standard operating procedure should be proposed for each organ after sufficient discussion is conducted between the surgeon and pathologists. In addition, we hope that our results will help to successfully perform the reimbursed CGP tests such as OncoGuide NCC Oncopanel System and FoundationOne CDx.

## AUTHOR CONTRIBUTIONS

*Conception and design of the study*: Hiroshi Nishihara, Emmy Yanagita. *Acquisition and analysis of data*: Emmy Yanagita, Hiroshi Yamada, Eriko Aimono, Kohei Nakamura. *Drafting the manuscript or figures*: Hiroshi Nishihara, Emmy Yanagita, Akira Hirasawa. *Statistical analysis*: Tetsuro Kobayashi.

## CONFLICT OF INTEREST STATEMENT

None declared.

## Supporting information


**Supplemental Figure 1** Sequence success rate according to DIN.

**Supplemental Figure 2** Sample distribution of “Success” cases and “Failure” cases according to DIN and DNA concentration.

**Supplemental Figure 3** ROC curves when DIN and DNA concentration are used in combination for predicting successful sequencing; using varying DIN values with DNA concentrations fixed at 10 ng/μl (A) and 20 ng/μl (B); and using varying DNA concentrations with DIN level fixed at 2.1 (C) and 2.5 (D).

**Supplemental Figure 4** Sequence result with DIN and DNA concentration of individual samples.

**Supplemental Table 1** The 160 genes list of GeneRead® Comprehensive Cancer Panel provided from QIAGEN.

**Supplemental Table 2** Sensitivity and specificity of DIN (A) and DNA concentration (conc.) (B) to predict successful sequencing. DNA conc.; ng/μl.

**Supplemental Table 3** Sensitivity and Specificity according to the DNA concentration (conc.) and DIN to predict successful sequencing.
