# Supplementary figures and images for: The DNA integrity number and concentration are useful parameters for successful comprehensive genomic profiling test for cancer using formalin‐fixed paraffin embedded tissue
Source: Pathol Int. 2023 Mar 27;73(5):198–206. doi: 10.1111/pin.13318 (PMC11551815; doi:10.1111/pin.13318)

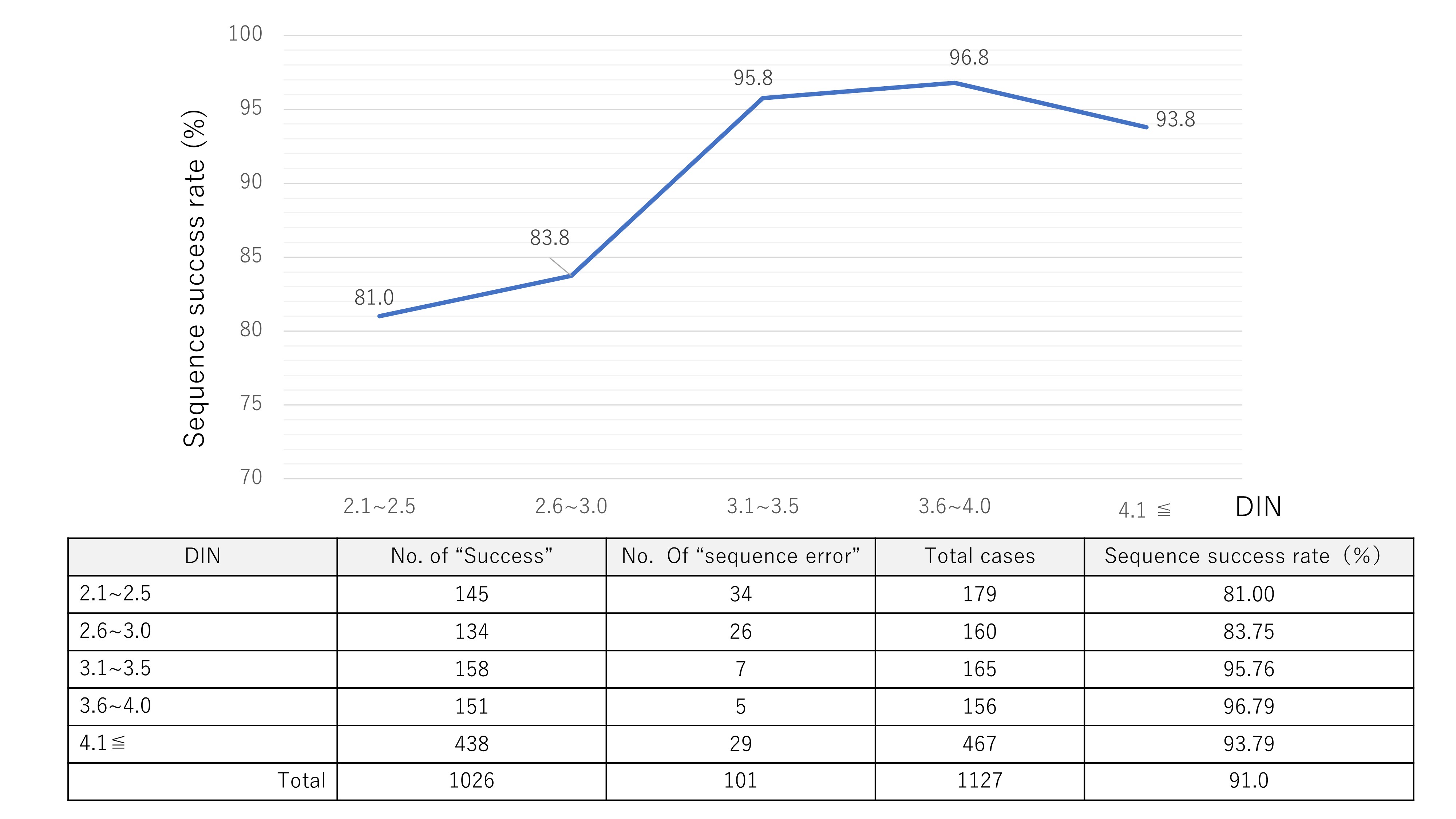

Supplement: Supplementary file 1 — Supplemental Figure 1 Sequence success rate according to DIN. [file PIN-73-198-s002.JPG]

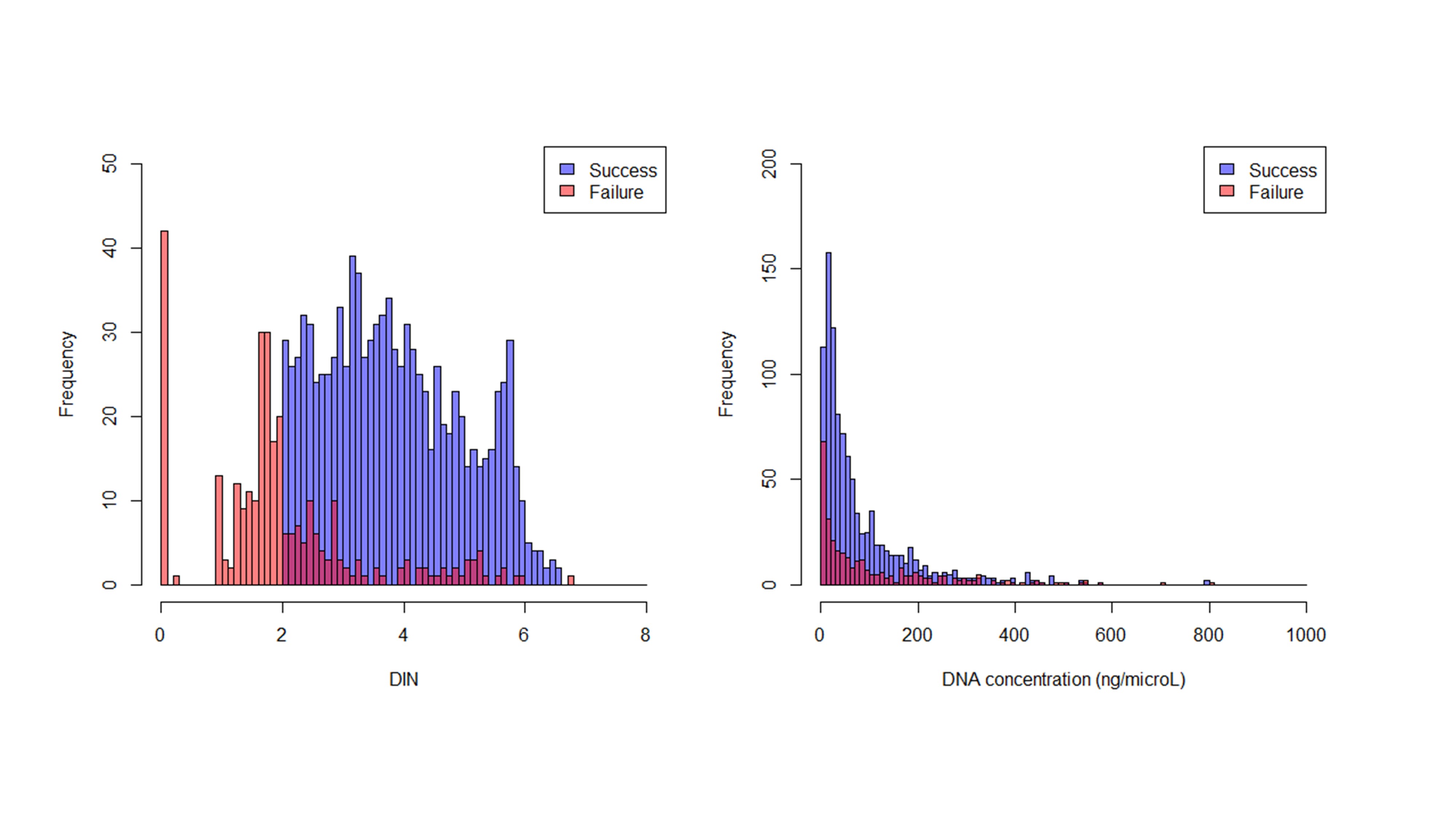

Supplement: Supplementary file 2 — Supplemental Figure 2 Sample distribution of “Success” cases and “Failure” cases according to DIN and DNA concentration. [file PIN-73-198-s007.JPG]

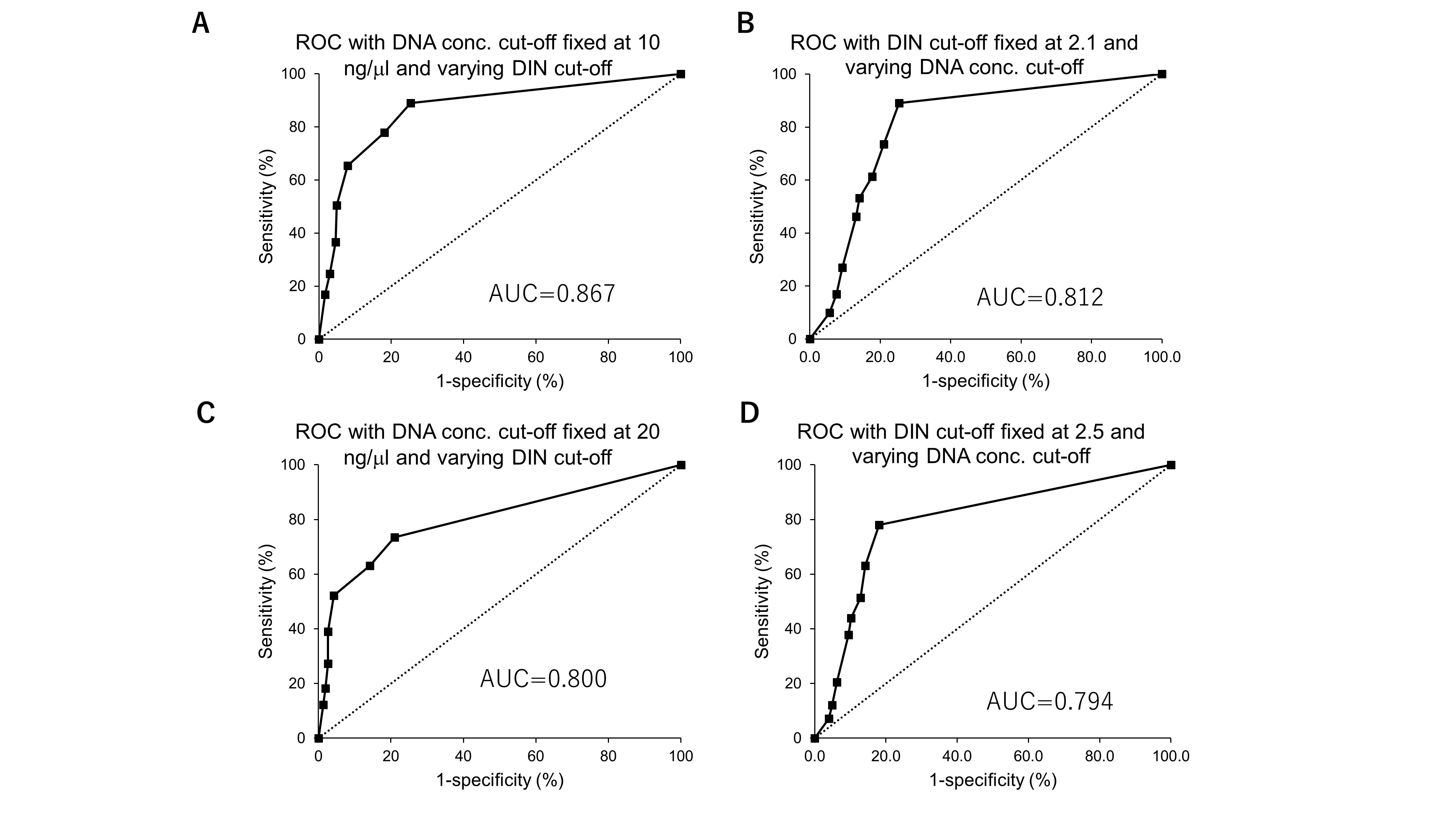

Supplement: Supplementary file 3 — Supplemental Figure 3 ROC curves when DIN and DNA concentration are used in combination for predicting successful sequencing; using varying DIN values with DNA concentrations fixed at 10 ng/μl (A) and 20 ng/μl (B); and using varying DNA concentrations with DIN level fixed at 2.1 (C) and 2.5 (D). [file PIN-73-198-s005.JPG]

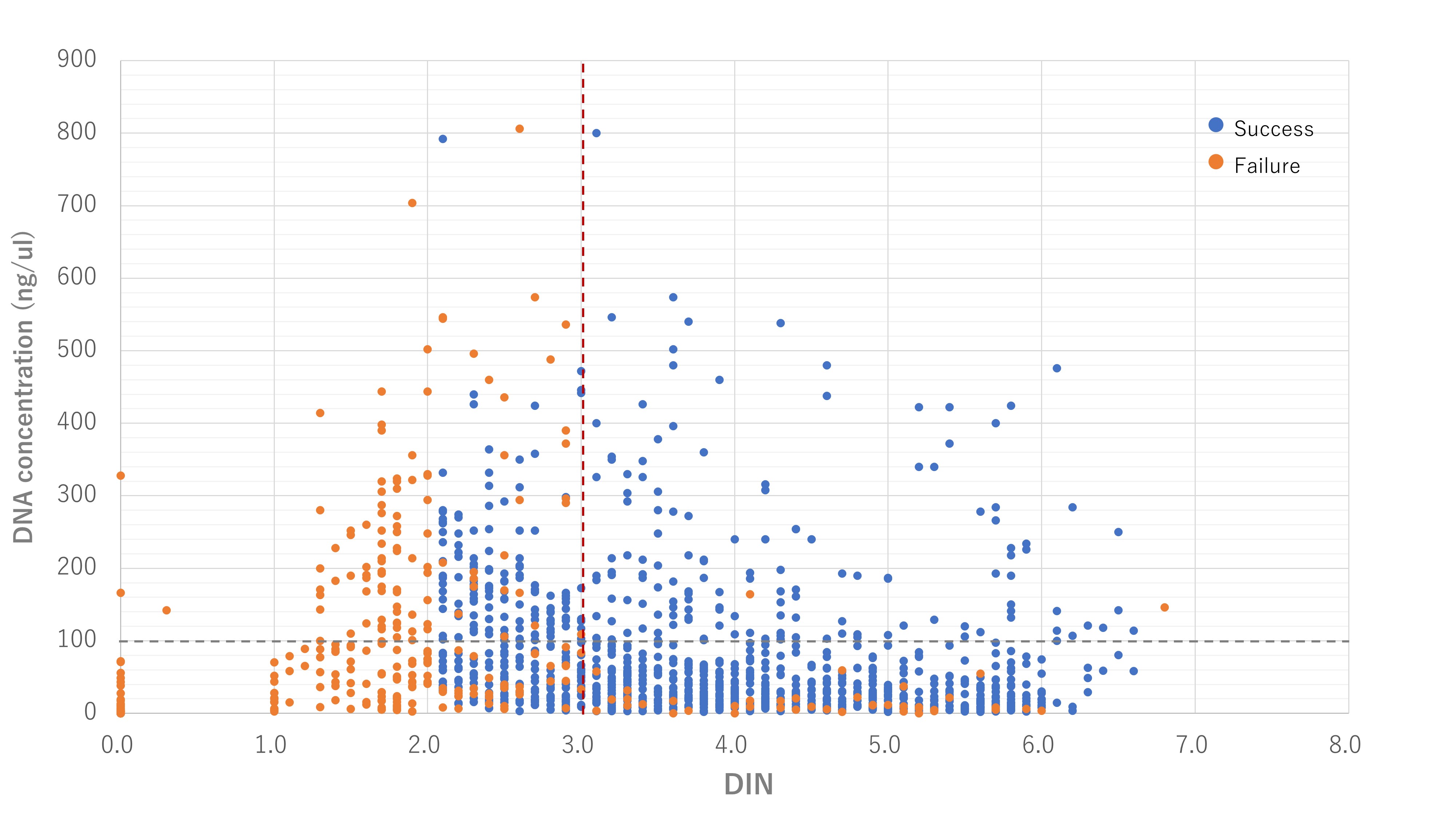

Supplement: Supplementary file 4 — Supplemental Figure 4 Sequence result with DIN and DNA concentration of individual samples. [file PIN-73-198-s004.JPG]
